# Research and data systems to promote equal access to postacute rehabilitation

**DOI:** 10.1186/2045-4015-2-28

**Published:** 2013-07-23

**Authors:** Linda Resnik

**Affiliations:** 1 Providence VA Medical Center, 830 Chalkstone Avenue, Providence, RI 02908, USA

## Abstract

The first national study in Israel of post-acute rehabilitation service use for elderly patients with stroke and hip fracture reported regional variation in care receipt. Although lower likelihood of admission to inpatient rehabilitation was observed in districts with known shortages of rehabilitation beds, supply alone did not explain the findings. This commentary explores other potential contributing factors. It argues that greater uniformity in the process and documentation of discharge planning in combination with decision support would help to standardize provider behavior. Implementation of a system of functional status data collection that is linked to administrative data is recommended to enable examination of the impact of care receipt and variation. Additional research is needed to provide a clearer understanding of factors contributing to regional variation and to identify solutions to ensure equal access to post-acute rehabilitation services in Israel.

## Background

In the current issue of the Israeli Journal of Health Policy and Research, Zucker and colleagues report the results of a nationwide study of post-acute rehabilitation service delivery to elderly persons after stroke and hip fracture [1]. Their study is the first to document the amount and variation in post-acute inpatient rehabilitation service use in a representative elderly Israeli sample. This work builds upon earlier research quantifying use of inpatient rehabilitation for all Israeli patients with stroke, which also found regional variation in post-acute rehabilitation [2]. In both studies, the geographic variation was attributed, in part, to the known shortage of rehabilitation beds.

Although prior studies show that availability of clinical services is a stronger predictor than patient characteristics when determining type of post-acute care [3], the data presented in Zucker’s paper strongly suggests that there are other contributing factors. If supply was the strongest driving factor in service use, we’d expect that patients residing in the North district would have the lowest likelihood of inpatient rehabilitation use for both diagnoses, and that the likelihood of receipt of care in the North would be lower than in Jerusalem because Jerusalem has greater availability of rehabilitation beds per person than does the North district (1/1268 elderly persons, compared to 1/3200 respectively).

However, Zucker found that, according to health plan data, patients living in Jerusalem had a 24% likelihood of receiving inpatient rehabilitation after hip fracture, and patients living in the North had a 48% likelihood of receipt as compared to patients living in the Center district. Likelihood of receipt of inpatient rehabilitation after stroke was lower only for patients living in Jerusalem, but not for patients living in the North as compared to those from the Center district. These findings contrast with earlier reports of lower likelihood of inpatient rehabilitation receipt for patients living in the North district, but not in Jerusalem [2]. This suggests that additional causes for geographic variation in care receipt for elderly patients are very important.

Other explanations for regional variation include socioeconomics, religious and cultural differences between regions of the country. Jerusalem is known to have poverty rates that are nearly twice the national average, as well as high rates of unemployment. Jerusalem also has a higher proportion of Ultraorthodox Jews and Arab residents than do other districts. The association between these factors and receipt of post-acute rehabilitation services for the elderly should be explored.

Only 11% of stroke and 14% of hip fracture patients had ambulatory rehabilitation services without prior inpatient rehabilitation. However, it is possible that additional patients were referred directly to ambulatory rehabilitation (because no inpatient bed was available, or because of provider or patient preference), but at four months post-stroke had not yet received them. Shortages and long waiting times at community based rehabilitation centers across Israel, especially for older persons with chronic disease have been reported [4]. Thus, further study is needed to determine whether patients who had not received any ambulatory rehabilitation services were on wait lists for treatment. Additional research is also needed to quantify regional variation in ambulatory services receipt. These types of study are critical to help identify needs and justify the placement of new inpatient and community based facilities as well as the expansion of rehabilitation beds and capacities within existing facilities.

It is also possible that there is regional variation in the provider referral process or practice preference with clinicians in Jerusalem and the North less likely to evaluate the rehabilitation needs of elderly patients and/or less likely refer them for post-acute care. In an effort to promote equal access to rehabilitation services, the Israeli Ministry of Health developed and disseminated referral regulations to acute care hospitals. However, the impact of these regulations is unknown. It was not possible for the Zucker study to determine whether rehabilitation needs were being assessed because the recommendations for rehabilitation were not consistently present in hospital discharge letters. Thus, the authors were not able to uniformly discern when providers had failed to evaluate and consider rehabilitation needs, when rehabilitation services were ordered but not received, or which type of services were ordered.

This information gap points to the need for re-engineering of hospital systems to ensure that discharge recommendations are uniformly documented using a standardized documentation template. Such a template should specify the provider’s evaluation of rehabilitation potential, and clearly indicate when a referral for services has been issued. This requirement, when combined with auditing and reporting of compliance, would drive uniformity in provider behavior.

Further, automated decision support can be implemented within the electronic health system to help providers make appropriate rehabilitation referrals. Such a decision support system should draw upon standard data elements collected during physical and occupational therapy encounters during the acute hospital stay. When clinicians have access to organized patient data, they are much more likely to refer patients to rehabilitation [5]. An example of a similar system to promote appropriate referral for acute rehabilitation services was recently implemented by the Cleveland Clinic Health System in the U.S. [6].

Early rehabilitation is important to maximize functional gain after stroke. Patients who are functionally dependent and who are discharged home without needed rehabilitation services are more likely to be readmitted to acute hospitalization. These patients will almost certainly incur higher societal costs in terms of need for paid or unpaid personal care. Additional research should be conducted to study the impact of variation of care receipt on patient outcomes and costs of care. In order to conduct this type of research across the country of Israel an organized system of outcome assessment is needed. Such a system would track patients’ functional status in the hospital and reassess patients as they transition to post-acute care and beyond. This type of data, embedded in the electronic health record, and merged with administrative data on service use and health care costs can be used to examine short and long-term outcomes of care. Israel’s Maccabi Health Plan has begun integrating functional status data collection with the electronic health record in the outpatient setting and using this data for quality improvement in the physical therapy service [7]. This type of effort could be expanded so that health outcome tracking begins in the acute care setting and includes appropriate metrics collected across all settings.

I commend the authors for conducting this important study which is the first to describe national patterns of post-acute rehabilitation service use for elderly patients after stroke and hip fracture in Israel. Their findings suggest a need for system wide improvements in data collection, as well as additional research to provide a deeper understanding of determinants and impact of regional variation. This type of effort is also needed in other countries, where geographic variation in rehabilitation service delivery has been documented.

While adding rehabilitation service capacity can be costly, the motivation to make this investment will be strengthened when there is evidence that links the receipt of inpatient and ambulatory rehabilitation to improved functional status, and lower long-term health care costs due to reductions in rehospitalization, and costs for personal care. This type of understanding will be needed to identify solutions to insure equal access to post-acute rehabilitation services in Israel.

## Competing interests

The author declares that she has no competing interests.

## Author information

Linda Resnik, PT, PhD is Associate Professor (Research) in the Department of Health Services, Policy and Practice, Brown University, Providence RI and a Research Health Scientist at the Providence VA Medical Center, Providence, RI.

## Commentary on the manuscript

Regional gaps in the provision of inpatient rehabilitation services for the elderly in Israel: Results of a national survey by Inbar Zucker^1*^, Irit Laxer^2^, Iris Rasooli^2^, Shulamit Han^2^, Aaron Cohen^2^, and Tamar Shohat^1^.

^1^Israel Center of Disease Control and the ^2^Geriatric Department of the Ministry of Health.
